# Stem cell: from basic theoretical assumptions and mathematical concepts to the computational models

**DOI:** 10.1186/1753-6561-5-S8-P108

**Published:** 2011-11-22

**Authors:** Katya Simeonova, Ganka Milanova

**Affiliations:** 1 Institute of Mechanics, Bulgarian Academy of Sciences, 1113 Sofia, Bulgaria; 2 University of Architecture, Civil Engineering and Geodesy, 1000 Sofia, Bulgaria

## Background

Stem cells (SC) and their therapeutic applications have a great promise for treatment of many human diseases, [1]. Characteristics of Embryonic Stem Cells (ESC) have been presented in [2]. The aim of the paper presented has been formulated as follows: to give some classical mechanics and mathematics theories for SC studies. Mathematical models for cancer diseases and neurological disorders have been discussed too.

## Materials and methods

Models, including a viscoelastic continuum, a combination of viscoelastic fluid have been developed as well, [3]. By relatively modern (for that time) microscopic techniques, developed during the eighteenth have been observed the images of living cells. Later using imaging mechanics, have been discovered AFM, imaging for study of living cells. High resolution of AFM imaging “has provided information on the structure. It has been proved as well, [4] that blood, blood vessels and nerves, could be tested mechanically.

## Mathematical models of SC for neuroscience

Theoretical concepts and mathematical models have been proposed for explanation quantitatively biological mechanisms. Mechanisms and models of cellular State Transition have been discussed too. Other periodic hematological diseases involve oscillations in all of the blood cells. In the paper [5], has been developed a simple mathematical model0. The probabilities for CSC to reach levels, compatible with the diagnosis of acute leukemia, by CSC, as well as probability for extinction have been computed analytically.

## An analytical model

The probability “that at least once, the population will have M_1_CSC, approximately given by:(1)

Here M_1_CSC, at time *t* = 0, *r* is the relative fitness parameter of CSC. The general expression for the fixation probability could be given as follows:(2)

For the birth-exports process authors in [11], considered the transition probability T*(*k*, *NSC* ), that numbers of CSC increasing from *k* to *k*+*1*, given by formula:(3)

## A computational model, results

Solving the equation (4) we obtained the next formula for *k*:(4)

**Figure 1:**
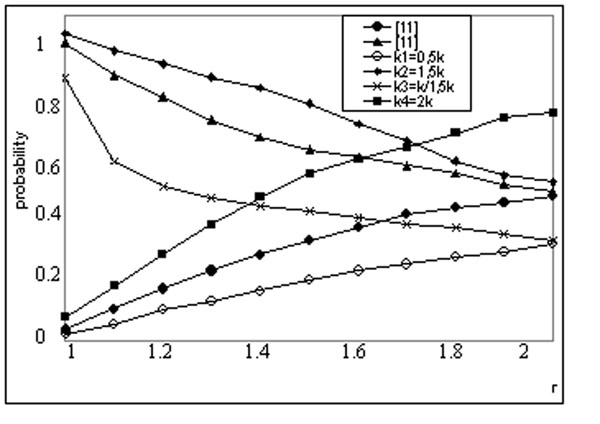
Curves, reflecting change of probability, versus stiffness parameter, at different values of *k.*

Here all parameters: *p*, *r* and *N* have been presented in the work [5]. So we investigate the effect of parameter *k*, on the probability *p_CSC_*(*k*). Numerical FORTRAN programs designed by authors have been used. By numerical experiments have been obtained dependencies at different *k*. Results, have been analyzed and shown (Figure 1).

## Conclusions

Future problems and current studies on SC are: SC is very promising tool for various biopharm applications, Future technologies will be enabled fuller understanding of SC, Future directions for human S Cell Culture optimization, ES could be used as vechile for gene therapy.
